# Vincristine-induced vocal cord palsy and successful re-treatment in a patient with diffuse large B cell Lymphoma: a case report

**DOI:** 10.1186/1756-0500-7-318

**Published:** 2014-05-27

**Authors:** Zarka Samoon, Munira Shabbir-Moosajee

**Affiliations:** 1 Department of Oncology, Aga Khan University Hospital, Stadium Road, PO Box: 3500, Karachi 74800, Pakistan

**Keywords:** Vincristine, Vocal cord palsy, Re-treatment

## Abstract

**Background:**

Vincristine, a type of vinca alkaloid, is widely used in the treatment of various childhood and adult malignancies. A well-known side effect of vincristine is its neurotoxicity and it is rarely indicted in vagus nerve involvement. Vincristine induced vocal cord palsy is a potentially reversible condition, with the mainstay of therapy being withdrawal of the offending drug. However, there are no clear guidelines regarding the possibility of re-treatment with the causative agent.

**Case presentation:**

A 58 year old Asian male presented with constipation and abdominal distension. Diagnostic investigations revealed stage IVB diffuse large B cell lymphoma (DLBCL). The patient was subsequently started on R-CHOP (Rituximab, Cyclophosphamide, Doxorubicin, Vincristine, and Prednisolone). On day twelve of receiving course four of R-CHOP, our patient presented to the hospital with a history of hoarseness of voice. Clinical and radiological examination revealed bilateral vocal cord palsy. Tracheostomy was done in view of a compromised airway. The patient subsequently went on to receive two more cycles of R-CHOP. Two weeks later Flexible laryngoscopy showed no lesion and the vocal cords were moving normally. The tracheostomy was removed. His voice has improved since and the patient is currently in remission.

**Conclusion:**

The occurrence of vincristine induced vocal cord palsy has been well reported in the literature. We strongly believe that our patient developed vocal cord palsy secondary to vincristine. The uniqueness of our patient’s case lies in successful re-treatment of our patient with the offending drug. To the best of our knowledge this is the third instance where a patient was successfully re-treated with vincristine after having developed vocal cord palsy as a result of its use.

## Background

Vincristine, a type of vinca alkaloid, is widely used in the treatment of various childhood and adult malignancies. Peripheral neuropathy is a well-known and recognized side effect of this drug. However, there atypical neurotoxic manifestations of vincristine, some of which may be fatal. Early recognition of such neurological complications and their prompt management may lead to complete recovery.

We present a case of a fifty eight year old man receiving R-CHOP for DLBCL who suddenly developed bilateral vocal cord palsy secondary to vincristine induced neurotoxicity. He had spontaneous recovery of neurological function despite continuation of the drug.

## Case presentation

**Figure 1:**
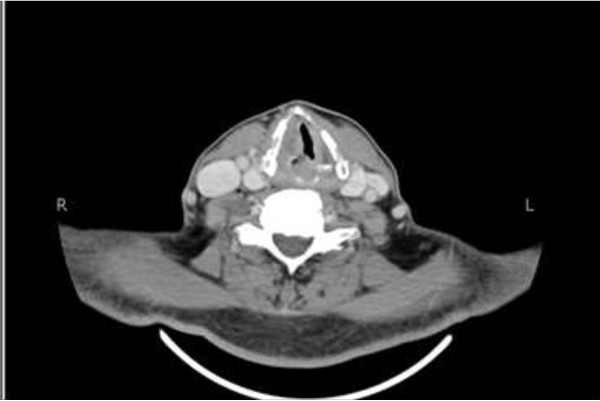
CT scan of the neck showing a hyper dense mass lesion involving right aryepiglottic fold extending into right vocal cord.

We report the case of a 58 year old man who presented in August 2012 with complaints of constipation and abdominal distension. His workup revealed stage IVB diffuse large B cell lymphoma (DLBCL). He was subsequently started on systemic chemotherapy with R- CHOP (Rituximab 375 mg/m^2^, Cyclophosphamide 750 mg/m^2^, Doxorubicin 50 mg/m^2^, Vincristine 1.8 mg/m^2^,, and Prednisolone 50 mg/m^2^,) with an excellent clinical response. He received four courses of R-CHOP uneventfully till November 2012. On day twelve of course four, the patient presented to the hospital with a history of hoarseness of voice, difficulty in breathing and cough. On physical examination the patient was found to have bilateral wheeze and stridor. The patient was not taking any antibacterial or antifungal drugs at that point in time. Laboratory work up was significant for neutropenia with an absolute neutrophil count (ANC) of 336. CT was performed, which showed a hyper dense mass lesion involving right aryepiglottic fold extending into right vocal cord, which was new and not present on the earlier scan (Figure 1). The remainder of the scan revealed significant improvement of overall disease process with substantial reduction in size of enlarged mesenteric lymph nodes and resolution of gross ascites along with non-visualization of diffuse omental nodularity, caking and retroperitoneal lymph nodes.

Fiber-optic laryngoscopy was performed, which showed swelling in the posterior part of right vocal cord, both vocal cords fixed with a narrow glottic opening. Tracheostomy was done in view of a compromised airway. Intra-operatively, the right vocal cord appeared inflamed and was biopsied. Biopsy revealed severe acute and chronic inflammation with no evidence of malignancy.

The patient was discharged from hospital and continued antibiotics outpatient. Subsequently flexible laryngoscopy showed left vocal cord moving normally and right vocal cord showing limited movement. The patient then received two more courses of R-CHOP till January 2013, with full dose of vincristine, as the main differential for vocal cord palsy was mainly infection. The vocal cords had not regained normal function till that point in time.

Two weeks after completion of therapy flexible laryngoscopy showed no lesion and the vocal cords were moving normally. The tracheostomy was removed. His voice has improved since. A positron emission tomogram done after six courses of R-CHOP show no residual disease and to date he remains disease free.

## Discussion

Rituximab with CHOP like chemotherapy is the standard of care in patients with diffuse large B cell lymphoma of which vincristine is an essential component [1,2]. Vincristine is known to cause various side effects such as constipation, hair loss, hyponatremia however neurotoxicity is the most common amongst them [3]. Vincristine is postulated to interfere with microtubule formation and cause axonal degeneration [4]. Factors leading to more severe neurotoxicity include hypersensitivity to the drug, pre-existing liver dysfunction, hereditary neuropathy, and concomitant use of certain drugs, such as allopurinol, erythromycin, isoniazid, mitomycin, phenytoin and itraconazole [5,6]. Vincristine induced neurotoxicity can present as a mixed sensorimotor neuropathy, autonomic dysfunction and less commonly cranial nerve involvement and encephalopathy. The cranial nerve involvement can be present as transient cortical blindness, hearing loss, facial palsy, occulomotor nerve dysfunction, jaw pain as a manifestation of trigeminal neuropathy, facial palsy and rarely recurrent laryngeal nerve palsy which can result in vocal cord palsy [7,8]. Vocal cord paralysis can be due to peripheral damage to the laryngeal nerve or to the vagus nerve nuclei and its origin within the central nervous system [9]. Bilateral involvement is usually due to involvement of the vagal nuclei while unilateral involvement is believed to be due to peripheral damage of the vagus nerve [10].

Vincristine induced vocal cord palsy has mainly been reported in the pediatrics age group, with few case reports in adult. Its prevalence is said to about 1.36% in pediatric patients [11]. From literature review it is evident that vincristine induced neuropathy is related to dose intensity along with cumulative dose effect [7]. In most patients, vincristine induced vocal cord palsy occurs beyond its second dose.

There is no specific treatment of vincristine induced vocal cord palsy. Pyridoxine and pyridostigmine has been associated with an early recovery (within 1–2 weeks) of vincristine induced peripheral neuropathy in a case series of patients [12]. The role of glutamic acid in decreasing vincristine induced neurotoxicity has been analyzed in some studies involving rat model [13], however this was not found to be effective when used in a patient with vincristine induced vocal cord palsy [14]. Overall about 55-66% of all pediatrics patients presenting with bilateral vocal cord paralysis require airway protection with a tracheostomy [8]. In a case series of 10 pediatrics patients with vincristine induced bilateral vocal cord palsy, only 2 required tracheostomy [11]. Vincristine induced vocal cord palsy may be reversible and complete recovery occurs in 6–9 months [6,7,15].

Besides our patient, there are only four other reported cases of reintroduction of vincristine at full dose [11,14,16,17] and in two of them there was a relapse [11,14,16] (Table 1). The reason for continuing vincristine at full dose in our case was that at that point our differential for vocal cord palsy was mainly infection, and the mass at the right aryepiglottic fold was a red herring. In retrospect, it was thought that the mass effect was likely a result of the paralyzed, fixed vocal cord causing distortion of the aryepiglottic fold. The patient had a tracheostomy for airway protection and chemotherapy was continued with vincristine at full dose. Interestingly, our patient regained vocal cord function despite continuation of vincristine and was successfully decanulated two weeks after completion of chemotherapy.

**Table 1:** Literature review of cases of Vincristine-Induced Laryngeal Paralysis (VLP) in whom full dose of vincristine was re-administered

**Authors**	**Diagnosis**	**Age/Sex**	**Manifestation**	**Airway intervention**	**Duration of paralysis**	**Administration of vincristine**
Ahmed et al. [16]	Acute lymphoblastic leukemia	2 yr/M	Bilateral VCP	No	6 months	Redeveloped hoarseness on receiving full dose. VCR Ommited
Kuruvilla et al. [11]	Rhabdomyosarcoma of testicles	3 yr/M	Left VCP	No	4 weeks	Gradually received complete dose.
Farrugia [14]	Acute lymphoblastic leukemia	18 months/F	Bilateral VCP	No	4 weeks	Redeveloped hoarseness on receiving full dose. Dose reduced to 1/3^rd^ subsequently
Burns et al. [17]	Hodgkin’s disease	48 yr/M	Left VCP	No	5 weeks	Received full dose uneventfully.

## Conclusion

In conclusion, we report a case of vincristine induced bilateral recurrent laryngeal nerve palsy who had full recovery of vocal cord palsy within two weeks after completion of definitive treatment of diffuse large B cell lymphoma. We think that vincristine induced vocal cord palsy is potentially reversible and careful protection of airway is needed when completion of treatment is the goal.

## Consent

Written informed consent was obtained from the patient for publication of this case report and any accompanying images. A copy of the written consent is available for review by the Editor-in-Chief of this journal.

## Competing interests

The authors declare that they have no competing interests

## Author’s contributions

ZS did the literature search and drafted the manuscript. MSM conceived the case report and helped in drafting the manuscript. Both authors read and approved the final manuscript.
